# Dosimetric effects of supine immobilization devices on the skin in intensity-modulated radiation therapy for breast cancer: a retrospective study

**DOI:** 10.1186/s12885-021-08119-6

**Published:** 2021-04-09

**Authors:** Ran Lv, Guangyi Yang, Yongzhi Huang, Yanhong Wang

**Affiliations:** Second Affiliated Hospital of Fujian Medical University, NO 950, Donghai Street, Fengze District, Quanzhou, 362000 Fujian China

**Keywords:** Supine immobilization devices, Breast cancer, Intensity-modulated radiation therapy, Skin dose

## Abstract

**Background:**

The dose perturbation effect of immobilization devices is often overlooked in intensity-modulated radiation therapy (IMRT) for breast cancer (BC). This retrospective study assessed the dosimetric effects of supine immobilization devices on the skin using a commercial treatment planning system.

**Methods:**

Forty women with BC were divided into four groups according to the type of primary surgery: groups A and B included patients with left and right BC, respectively, who received 50 Gy radiotherapy in 25 fractions after radical mastectomy, while groups C and D included patients with left and right BC, respectively, who received breast-conservation surgery (BCS) and 40.05 Gy in 15 fractions as well as a tumor bed simultaneous integrated boost to 45 Gy. A 0.2-cm thick skin contour and two sets of body contours were outlined for each patient. Dose calculations were conducted for the two sets of contours using the same plan. The dose differences were assessed by comparing the dose-volume histogram parameter results and by plan subtraction.

**Results:**

The supine immobilization devices for BC resulted in significantly increased skin doses, which may ultimately lead to skin toxicity. The mean dose increased by approximately 0.5 and 0.45 Gy in groups A and B after radical mastectomy and by 2.7 and 3.25 Gy in groups C and D after BCS; in groups A–D, the percentages of total normal skin volume receiving equal to or greater than 5 Gy (V_5_) increased by 0.54, 1.15, 2.67, and 1.94%, respectively, while the V_10_ increased by 1.27, 1.83, 1.36, and 2.88%; the V_20_ by 0.85, 1.87, 2.76, and 4.86%; the V_30_ by 1.3, 1.24, 10.58, and 11.91%; and the V_40_ by 1.29, 0.65, 10, and 10.51%. The dose encompassing the planning target volume and other organs at risk, showed little distinction between IMRT plans without and with consideration of immobilization devices.

**Conclusions:**

The supine immobilization devices significantly increased the dose to the skin, especially for patients with BCS. Thus, immobilization devices should be included in the external contour to account for dose attenuation and skin dose increment.

**Trial registration:**

This study does not report on interventions in human health care.

## Background

Breast immobilization devices are commonly used in radiation oncology to provide breast cancer (BC) patients support and improve positional reproducibility during their fractionated radiotherapy [1–3]. In actual clinical practice, the beam attenuation and build-up perturbation effect caused by immobilization devices are often overlooked because the carbon fiber materials widely used in these devices are believed to be radio translucent for mega-voltage photons [4]. However, the density of carbon fiber is not equivalent to air; thus, attenuation and scattering can occur when the radiation beams pass cross these immobilization systems [5, 6]. Previous studies have reported that immobilization devices used in radiotherapy reduced the tumor dose, increased the skin dose (bolus effect), and altered the dose distributions [7–9]. De Puysseleyr and colleagues reported that irradiating through carbon fiber immobilization devices for prone breast radiotherapy resulted in considerable beam attenuation (range: 5.33 to 7.57%) and degradation of skin sparing [7]. For Chinese BC patients, due to their small and compact breast glands, supine positioning remains the most common approach and has multiple advantages, including methodological simplicity, comfort and accuracy, reproducible positioning, and reduced mean dose to the heart [10, 11].

Compared to conventional wedge-based breast radiotherapy, intensity-modulated radiation therapy (IMRT) can deliver highly conformal and homogeneous dose distributions to targets and, thus, significantly decrease clinical toxicities such as dermatitis and edema [12–14]. These advantages have substantially increased the adoption of IMRT during breast radiotherapy [15, 16]. However, the increased beams and monitor units (MUs) have an increased propensity to deliver radiation beams through the immobilization devices, resulting in radiation immobilization device attenuation, ultimately compromising the target coverage and organ-at-risk (OAR) protection [17]. However, no study has yet assessed the dosimetric effects of supine breast immobilization devices on the delivered doses to the target volume and OARs for breast IMRT. Thus, this study quantified the dosimetric effect of supine immobilization devices by comparing the dose distributions calculated with and without breast immobilization devices and investigated the potential skin sparing for BC patients achievable with 6 MV photon beams in IMRT plans.

## Methods

### Patient data and setup

This study enrolled 40 women with BC who received adjuvant radiotherapy in our institution. The participants were divided into four groups according to the lesion location, type of primary site surgery, and irradiation field. Patients with left or right BC receiving radical mastectomy and chest plus infra/supraclavicular lymph node irradiation were assigned into groups A and B respectively. To ensure the same irradiation fields, the patients in these two groups had four or more involved lymph nodes and required infra/supraclavicular lymph node irradiation. In the same way, patients with left or right BC receiving breast-conservation surgery (BCS) and breast without lymph region irradiation were divided into groups C or D, respectively. No metastatic lymph nodes were detected in these two group patients and only the breast was irradiated. The patients’ ages ranged from 32 to 65 years, with a median age of 47 years.

### Simulation

**Fig. 1:**
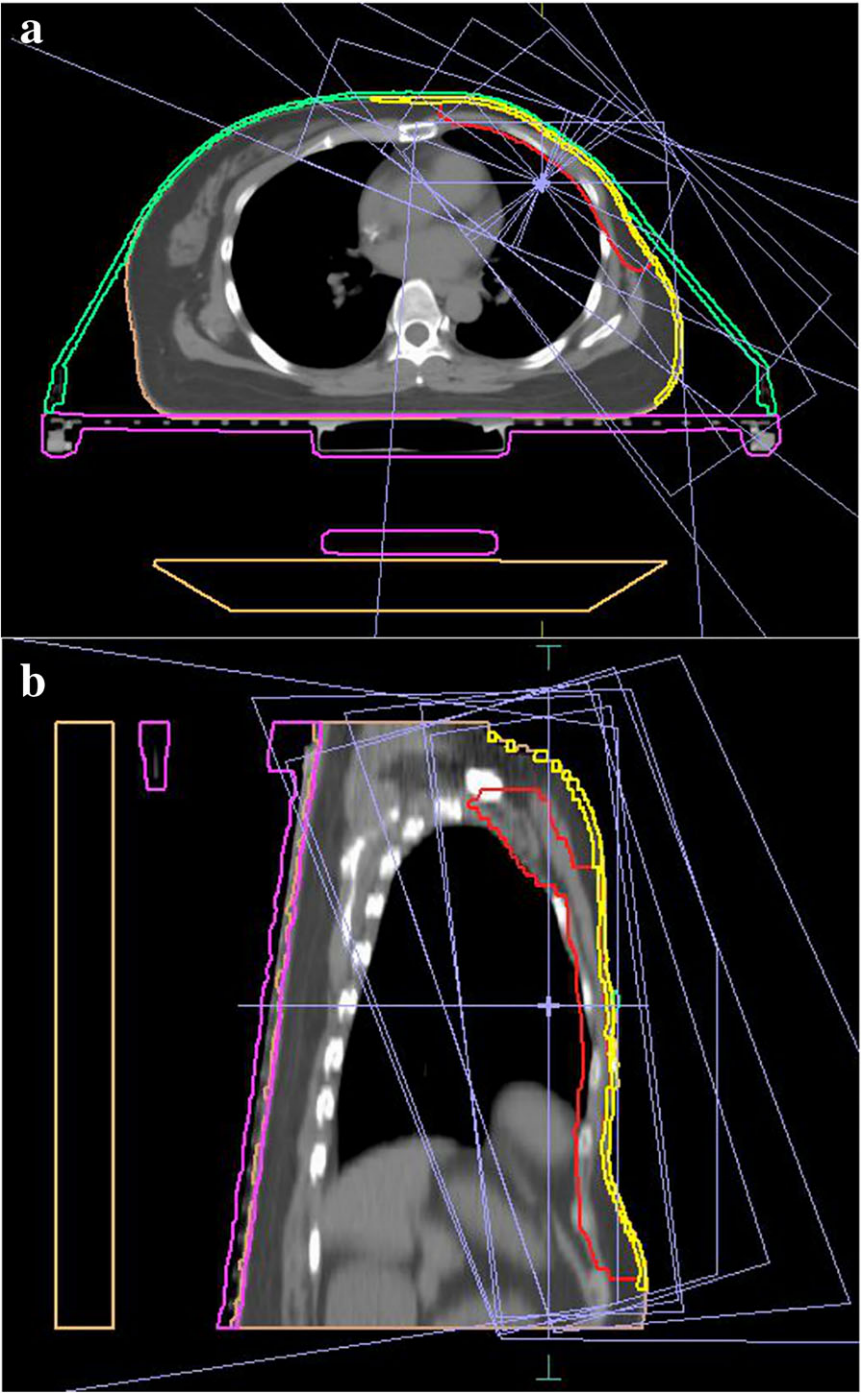
Display of the immobilization devices in the axial (**a**) and sagittal (**b**) views. The orange portion is the couch, the purple portion is the breast-board, the green portion is the chest fixation mask of thermoplastic, the skin contour is displayed in yellow, and the PTV is red

All patients were simulated in the head-first supine position using a carbon fiber breast bracket (Klarity Inc., Guangzhou, China) for body immobilization. The supporting board was inclined at 7°, 12°, 17°, or 23° to assure that the sternum was horizontal. The patients’ heads were positioned straight on a circle sponge head support, with the chin slightly upwards, avoiding skin folds at the lower neck. Both arms were raised over their heads using a pair of arm supports to adequately expose the breast, as well as a knee support to prevent the body from sliding down. A thermoplastic film (electron density 0.3–0.7, thickness 2.4 mm) (Klarity Medical Products, Newark OH) was custom-molded over the chest and attached to the bracket with a plastic batten (electron density 1–1.1). Computed tomography (CT) imaging with a 3-mm slice thickness was performed using a large-aperture CT simulation scanner (Brilliance, Philips Medical System, Amsterdam, Netherlands) (Fig.1). The scan range was from the first cervical vertebra to the diaphragm. The simulation CT images were transferred to the treatment planning system (TPS, Monaco V5.11, Elekta AB, Stockholm, Sweden) for target and OAR delineation and treatment planning.

### Regions of interest (ROIs)

The ROIs were delineated on CT images with the CT data set as soft tissue (window 600, level 40) by experienced oncologists according to the recommendation of the International Commission on Radiation Units and Measurements (ICRU Reports 83). For patients who underwent BCS, the clinical target volumes (CTVs) included all mammary glandular tissue and a CTV boost (the tumor bed including the clips and seroma plus a 5-mm margin in all directions without exceeding the CTV of the breast). The corresponding planning target volumes (PTVs) were generated by uniformly expanding 5 mm from the CTVs. Given the smaller mammary glands of Eastern women, the ventral border was placed 2 mm below the skin surface [18]. For patients with modified radical mastectomy, the CTV included the chest wall and infra/supraclavicular lymph nodes, with the ventral border next to the skin surface. The OARs, including the heart, left anterior descending artery (LAD), left ventricle (LV), contralateral breast, ipsilateral lung, and contralateral lung, were accounted for in left-side BC, while the liver was especially accounted for in right-side BC. The thyroid, larynx, esophagus, and spinal cord were also considered in patients receiving lymph node irradiation. To assess the surface dose variance from immobilization devices in TPS, the skin contour in this study was especially delineated in the treatment region with 2 mm thickness below the skin surface for each patient [Fig.1] [19].

For commercial TPS, an external structure, which should contain the materials involved in the calculation, must be defined to calculate the dose distributions. In this study, two sets of external body contours were created for each patient: one set included only the patient’s body without immobilization devices, while the other set included the patient’s external body contours and the whole breast immobilization device.

### Treatment planning and dose calculation

The prescription doses to the PTV boost and PTV breast were 45 Gy and 40.05 Gy, respectively, with a total of 15 fractions in the case of BCS. For mastectomy, the prescription was 50 Gy in 25 fractions. All patients were planned on a Monaco TPS at 6 MV using the dynamic inverse IMRT technique. Multi-beam IMRT employs three groups of similarly opposed lateral fields spaced through a 290–150° sector for left-side tumors and a 200–60° sector for right-side tumors around the target volume, which includes the breast/chest wall and regional nodes, as indicated in Fig.1. A 0° field was added when the periclavicular node region was included. A 0.5-cm bolus was added to the surface skin in the treatment region for patients with radical mastectomy to compensate for the build-up effect of X-rays; in contrast, a bolus was avoided for patients with breast-conserving surgery to improve the cosmetic outcome. The optimization was performed using the Monaco’s build-in Monte Carlo (MC) algorithm combined with Dynamic Multi-Leaf Collimator (DMLC) technology. The maximum and minimum doses were planned in accordance with the ICRU 83 recommendations, with dose constraints following the QUANTEC directive [19–21].

Two IMRT plans were generated for each patient, with plans not including the immobilization devices in the calculations defined as Plan- and dose distributions recalculated with the external body contour containing the immobilization device under the same irradiation constraints defined as Plan+.

### Statistical analysis

Dose-volume histograms (DVHs) are the popular method to evaluate the dose coverage of PTVs and OARs. For the PTVs in the present study, the parameters were the mean dose (D_mean_), the homogeneity index (HI), and the conformity index (CI). The HI and CI were respectively calculated as follows [22, 23].

1 $$ HI=\frac{D_{5\%}}{D_{95\%}} $$

2 $$ CI=\frac{TV1}{TV}\ast \frac{TV1}{VR1} $$

In formula (1), D_5%_ and D_95%_ were the doses received by 5 and 95% of the ROI volume, respectively. A HI value closer to 1 indicates a better uniformity of the dose distribution in the target volume. In formula (2), TV1 is the target volume that receives the prescription dose while TV is the target volume. VR1 is the total volume within the prescription isodose curve. The CI value ranges between 0 and 1, with higher values indicating better dose conformity. Regarding OARs, the average dose D_mean_, as well as the maximum dose (D_max_) and the dose-volume were calculated.

For each patient, the dosimetric effects due to the immobilization devices were calculated by plan subtraction in the TPS. $\overline{D}$ represented the average of parameter differences between Plan+ and Plan-, as shown in formula 3, while $\overline{D}$_%_ represents the average of the relative differences between Plan+ and Plan-.

3 $$ \overline{D}={\sum}_1^{10}\left[\left( plan+\right)-\left( plan-\right)\right]/10 $$

4 $$ \overline{D}\%={\sum}_1^{10}\left\{\left[\left( plann+\right)-\left( plann-\right)\right]\ast 100/\left( plann+\right)\right\}/10 $$

IBM SPSS Statistics for Windows, version 22.0 (IBM Corp., Armonk, NY, USA) was used to analyze all data. Wilcoxon matched-paired signed-rank tests were used to evaluate the significance of the observed differences between Plan+ and Plan-. The differences were considered statistically significant when *p* < 0.05.

## Results

**Fig. 2:**
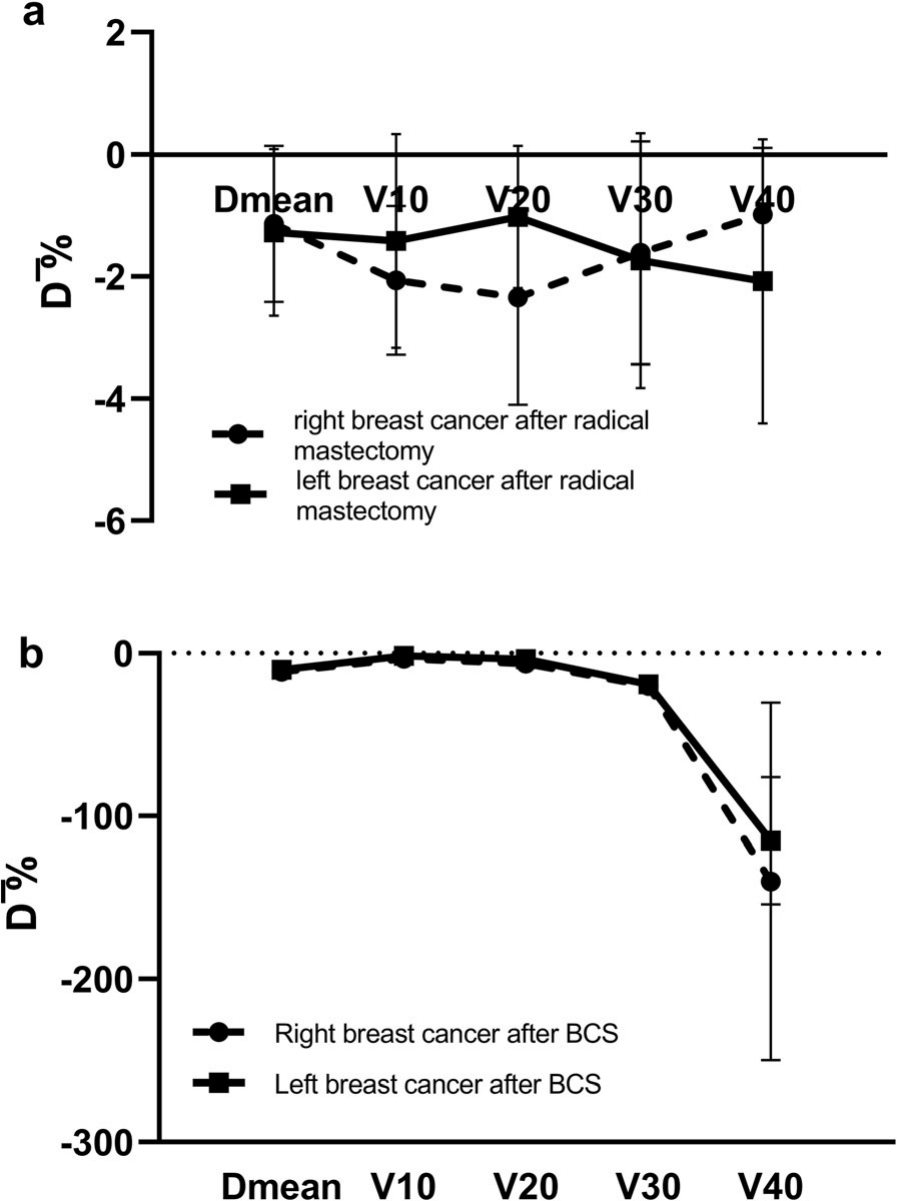
The $\overline{D}$ % of skin dosimetries for breast cancer after radical mastectomy (**a**) and BCS (**b**). The error bars reflect the standard error of the mean (r/ √n). The lines are drawn only to guide the eye

The comparisons of dosimetric differences between Plan+ and Plan- for BC patients receiving radical mastectomy are presented in Tables 1 and 2. The parameters (Coverage Index, D_mean_, D_2%_, and CI) of the PTV showed little difference, except for the HI of left-side cancer ($\overline{D}$ = − 0.006) and D_98%_ of right-side cancer ($\overline{D}$ = − 0.38 Gy) with statistically insignificant differences. Due to the bolus effect of the breast immobilization devices, the mean dose and relative volumes of skin receiving 5, 10, 20, 30, and 40 Gy were significantly increased for Plan+ ($\overline{D}$_and_ $\overline{D}$_%_ of 0.50 Gy and 1.25, 0.54 and 0.56%, 1.27 and 1.37%, 0.85 and 1.00%, 1.30 and 1.67%, and 1.29 and 1.99% for left BC and 0.45 Gy and 1.11, 1.15 and 1.20%, 1.83 and 2.01%, 1.87 and 2.27%, 1.24 and 1.56%, and 0.65 and 0.96% for right BC, respectively, all *p* < 0.05, Fig. 2a). However, there was no statistically significant difference in other OARs, except for the V_5_ of the larynx for right BC ($\overline{D}$_= 6.94 Gy, *p* = 0.014)_.

**Table 1:** Dosimetric parameters of PTV and OARs for 10 cases of left breast cancer after radical mastectomy

Parameter	Plan+	Plan-	$\overline{D}$(95%CI)	*p*
PTV				
Coverage Index	94.49 ± 1.08	95.01 ± 0.43	−0.52(− 1.18, 0.15)	0.112
D_2%_	54.14 ± 0.92	54.60 ± 0.45	−0.46(− 1.12, 0.19)	0.145
D_98%_	46.59 ± 1.92	47.07 ± 1.69	−0.48(− 1.13, 0.16)	0.124
D_mean_	52.46 ± 0.36	52.51 ± 0.31	−0.05(− 0.19, 0.10)	0.501
HI	1.09 ± 0.02	1.08 ± 0.01	0.006(0.001,0.011)	0.024
CI	0.65 ± 0.21	0.65 ± 0.22	0(−0.009, 0.005)	0.555
Skin				
D_mean_	40.39 ± 3.73	39.89 ± 3.76	0.50(0.11, 0.89)	0.018
V_5_	98.18 ± 1.78	97.64 ± 2.14	0.54(0.12,0.97)	0.018
V_10_	94.28 ± 3.77	93.01 ± 4.52	1.27(0.16, 2.49)	0.030
V_20_	85.71 ± 7.46	84.86 ± 7.61	0.85(0.17, 1.53)	0.020
V_30_	77.55 ± 8.79	76.25 ± 8.71	1.30(0.21, 2.40)	0.025
V_40_	64.91 ± 11.61	63.62 ± 11.51	1.29(0.15, 2.43)	0.031
Left Lung				
D_mean_	15.32 ± 1.627	15.36 ± 1.82	−0.03(−0.42, 0.35)	0.845
V_5_	61.90 ± 5.15	62.11 ± 4.99	−0.21(− 1.84, 1.43)	0.783
V_10_	44.11 ± 4.43	43.84 ± 4.90	0.26(− 1.14, 1.66)	0.682
V_20_	29.12 ± 3.85	29.25 ± 4.35	−0.14(−1.16, 0.88)	0.767
V_30_	19.98 ± 4.01	20.19 ± 4.31	−0.21(− 0.91, − 0.49)	0.514
Right Lung				
D_mean_	0.94 ± 0.26	0.94 ± 0.23	−0.001(− 0.05,0.05)	0.963
V_5_	20.08(14.30,38.73)	20.56(13.37,40.21)	−0.08(− 0.37,0.20)	0.515
V_15_	0(0,0.01)	0(0,0.025)	−0.01(− 0.02,0.01)	0.235
Heart				
D_mean_	7.45 ± 1.55	7.21 ± 1.40	0.24(−0.09, 0.57)	0.134
D_max_	51.74 ± 7.58	51.64 ± 7.71	0.11(−0.32, 0.53)	0.589
V_5_	58.64 ± 6.75	56.41 ± 7.87	2.23(−1.40,5.87)	0.198
V_25_	3.60 ± 3.61	3.56 ± 3.63	0.04(−0.10,0.19)	0.526
LAD				
D_mean_	37.93 ± 15.14	38.00 ± 14.86	−0.06(−0.73, 0.61)	0.839
D_max_	3.51 ± 3.41	3.45 ± 3.38	0.06(−0.07, 0.19)	0.319
V_5_	100(98.32,100)	100(98.14,100)	− 0.17(−5.72,5.38)	0.948
V_30_	13.48(0,45.85)	12.91(0,46.0)	0.36(− 0.09,0.80)	0.103
V_40_	1.91(0,34.55)	1.93(0,34.63)	−0.46(−1.70,0.79)	0.428
Left Vessile				
D_mean_	20.65 ± 10.30	20.65 ± 10.38	0(−3.07,3.07)	0.998
V_5_	72.82 ± 13.30	69.63 ± 16.16	3.19(−1.01,7.39)	0.120
V_23_	5.62 ± 5.53	5.65 ± 5.66	−0.03(− 0.27,0.21)	0.770
Right breast				
D_mean_	2.14 ± 1.13	2.21 ± 1.14	−0.07(− 0.27, 0.13)	0.448
D_2%_	14.76 ± 10.39	15.52 ± 10.61	−0.76(−2.24,0.72)	0.275
V_5_	8.62 ± 6.62	9.32 ± 6.78	−0.70(−2.14,0.74)	0.299
Spinal cord				
D_max_	17.17 ± 5.58	17.53 ± 6.25	−0.36(− 1.43,0.71)	0.464
D_2%_	13.03 ± 4.56	13.25 ± 4.56	−0.22(− 0.75,0.31)	0.375
Esophagus				
D_max_	53.60 ± 3.58	53.73 ± 2.83	−0.14(−1.02,0.75)	0.736
V_5_	59.22 ± 18.83	54.55 ± 18.17	4.67(−4.03,13.37)	0.256
Thyroid				
D_mean_	32.14 ± 3.92	32.27 ± 3.85	−0.13(− 0.63,0.38)	0.593
D_max_	54.68 ± 0.75	54.77 ± 1.16	−0.09(− 0.70,0.52)	0.752
V_5_	99.68 ± 0.79	99.56 ± 0.83	0.12(−0.31,0.54)	0.554
Larynx				
D_max_	42.62 ± 8.54	42.72 ± 8.29	−0.10(−1.33,1.13)	0.858
V_5_	90.05 ± 13.60	88.24 ± 15.09	1.81(−1.41,5.04)	0.235

**Table 2:** Dosimetric parameters of PTV and OARs for 10 cases of right breast cancer after radical mastectomy

Parameter	Plan+	Plan-	$\overline{D}$(95%CI)	*p*
PTV				
Coverage Index	94.51 ± 1.28	95.14 ± 0.80	−0.63(− 1.26, 0)	0.050
D_mean_	52.06 ± 0.20	52.18 ± 0.19	−0.12(− 0.30, 0.06)	0.174
D_2%_	53.97 ± 0.31	54.10 ± 0.30	−0.13(− 0.36, 0.09)	0.208
D_98%_	47.69 ± 1.18	48.07 ± 0.97	−0.38(− 0.67, − 0.09)	0.017
HI	1.08 ± 0.01	1.08 ± 0.01	0(−0.003,0.003)	1.000
CI	0.76 ± 0.04	0.75 ± 0.04	0.01(−0.002, 0.014)	0.140
Skin				
D_mean_	39.05 ± 3.39	38.61 ± 3.19	0.45(0.08, 0.81)	0.022
V_5_	97.87 ± 2.16	96.72 ± 3.19	1.15(0.28,2.02)	0.015
V_10_	91.29 ± 4.15	89.46 ± 4.37	1.83(1.07, 2.59)	0.000
V_20_	82.01 ± 6.91	80.14 ± 6.74	1.87(0.88, 2.86)	0.002
V_30_	74.16 ± 9.17	72.92 ± 8.25	1.24(0.21, 2.27)	0.023
V_40_	63.64 ± 9.97	62.99 ± 9.56	0.65(0.09, 1.21)	0.028
Right Lung				
D_mean_	14.65 ± 2.30	14.57 ± 2.43	0.07(−0.17, 0.32)	0.508
V_5_	62.10 ± 11.29	60.70 ± 10.80	1.40(−0.15, 2.96)	0.072
V_10_	41.97 ± 8.43	41.16 ± 8.64	0.82(− 0.21, 1.84)	0.105
V_20_	26.62 ± 5.38	26.78 ± 6.16	−0.16(− 0.91, 0.59)	0.636
V_30_	18.65 ± 3.96	18.84 ± 4.42	−0.19(− 0.69, 0.32)	0.427
Left Lung				
D_mean_	0.87 ± 0.26	0.86 ± 0.25	0.01(−0.01, 0.02)	0.485
V_5_	0.01(0,1.12)	0.02(0,1.15)	−0.01(− 0.05,0.03)	0.605
V_15_	0	0	0	
Heart				
D_mean_	2.79 ± 1.11	2.83 ± 1.05	−0.04(−0.20, 0.12)	0.592
D_max_	17.06 ± 6.93	17.17 ± 7.21	−0.11(−1.09, 0.88)	0.815
V_5_	17.86 ± 12.33	18.36 ± 11.95	−0.50(−2.32,1.31)	0.546
V_15_	0.01(0,0.34)	0.01(0,0.26)	−0.06(− 0.24,0.11)	0.419
Liver				
D_mean_	7.93 ± 2.55	7.90 ± 2.37	0.03(−0.23, 0.29)	0.787
V_5_	53.01 ± 17.55	53.22 ± 16.90	−0.21(−2.36,1.94)	0.831
V_13_	16.57 ± 8.27	16.36 ± 7.38	0.21(− 0.90,1.32)	0.675
Left breast				
D_mean_	1.89 ± 1.00	1.91 ± 1.03	−0.02(− 0.09, 0.05)	0.534
D_2%_	9.66(5.28,15.69)	9.74(5.66,15.55)	−0.02(− 0.37,0.34)	0.916
V_5_	8.02 ± 5.61	9.21 ± 6.44	−1.20(−3.64,1.25)	0.298
Spinal cord				
D_max_	18.91 ± 7.17	19.04 ± 7.54	−0.13(− 0.82,0.56)	0.685
D_2%_	15.04 ± 6.63	15.36 ± 6.56	−0.32(− 0.85,0.20)	0.194
Esophagus				
D_max_	51.26(46.71,52.98)	51.47(47.40,52.91)	−0.12(− 0.91,0.67)	0.739
V_5_	29.65(26.91,38.75)	30.88(24.54,35.82)	0.64(−1.09,2.36)	0.428
Thyroid				
D_mean_	38.19 ± 5.35	38.13 ± 5.13	0.06(−0.26,0.39)	0.681
D_max_	54.05 ± 0.64	54.41 ± 0.53	−0.35(− 0.94,0.23)	0.206
V_5_	100	100	0	
Larynx				
D_max_	49.57(34.50,51.60)	49.56(36.21,51.63)	−1.65(−5.35,2.04)	0.338
V_5_	79.41(46.83,100)	71.87(29.53,99.17)	6.94(1.74,12.14)	0.014

For patients with breast-conserving surgery, the dosimetric effects of immobilization devices were also calculated between Plan+ and Plan-. As shown in Tables 3 and 4 and Fig. 2b, the plans calculation with breast immobilization devices showed higher mean skin doses ($\overline{D}$_%_ = 9.07 ± 1.60% for left BC and 10.21 ± 2.95% for right BC) and higher volumes of skin receiving dose (5–40 Gy) radiation ($\overline{D}$_%_ 2.91, 1.65, 3.57, 15.85, and 51.86% for left BC and 2.05, 3.17, 5.84, 16.49 and 51.63% for right BC, respectively, Fig. 2b), which again displayed the bolus effect of immobilization devices. For left-side BC patients, the mean dose and relative irradiation volume of 5-30Gy of the left lung decreased, with little clinically significance, in Plan+ ($\overline{D}$-0.21 Gy for D_mean_, − 1.18% for V_5_, − 0.98% for V_10_, − 0.47% for V_20_, and − 0.28% for V_30_, respectively). Regarding PTV, the coverage index, HI, and CI were also altered, with statistical but not clinical significance. The other OARs far from the PTV, such as the contra-lung, heart, LV, LAD, and liver, showed non-significant differences between the two plans.

**Table 3:** Dosimetric parameters of PTV and OARs for 10 cases of left breast cancer after breast-conserving surgery

Parameter	Plan+	Plan-	$\overline{D}$(95%CI)	*p*
PGTV				
Coverage Index	97.02 ± 1.51	96.37 ± 1.52	0.65(− 0.24, 1.54)	0.133
D_mean_	46.99 ± 0.23	46.96 ± 0.18	0.03(− 0.15, 0.20)	0.726
HI	1.06 ± 0.01	1.07 ± 0.01	− 0.01(− 0.01, 0)	0.024
CI	0.66 ± 0.09	0.66 ± 0.10	0.01(− 0.01, 0.03)	0.460
PTV				
Coverage Index	95.64 ± 1.36	94.58 ± 0.69	1.06(0.18, 1.95)	0.024
D_mean_	43.43 ± 0.53	43.36 ± 0.66	0.07(−0.12, 0.26)	0.417
HI	1.18 ± 0.02	1.19 ± 0.02	−0.01(− 0.01, 0)	0.045
CI	0.76 ± 0.04	0.78 ± 0.04	−0.02(− 0.03, − 0.01)	0.001
Skin				
D_mean_	29.88 ± 2.81	27.18 ± 2.68	2.70(2.34, 3.07)	0.000
V_5_	91.28 ± 4.40	88.60 ± 4.26	2.67(1.58,3.76)	0.000
V_10_	83.70 ± 6.45	82.34 ± 6.68	1.36(0.95, 1.77)	0.000
V_20_	77.31 ± 8.22	74.57 ± 8.25	2.73(2.12, 3.34)	0.000
V_30_	67.66 ± 7.70	57.09 ± 7.97	10.58(9.17, 11.99)	0.000
V_40_	19.91 ± 9.64	9.91 ± 5.84	10.00(6.64, 13.36)	0.000
Left Lung				
D_mean_	7.82 ± 1.26	8.03 ± 1.23	−0.21(− 0.34, −0.08)	0.006
V_5_	34.86 ± 6.04	36.04 ± 5.29	−1.18(−2.33, − 0.03)	0.045
V_10_	25.27 ± 5.08	24.25 ± 4.76	−0.98(− 1.59, − 0.37)	0.005
V_20_	15.50 ± 3.49	15.97 ± 3.54	−0.47(− 0.76, − 0.18)	0.005
V_30_	9.36 ± 2.51	9.63 ± 2.54	−0.28(− 0.49, − 0.06)	0.019
Right Lung				
D_mean_	0.54 ± 0.22	0.53 ± 0.21	0.01(−0.01, 0.03)	0.423
V_5_	0.02(0,0.38)	0.01(0,0.51)	−2.54(−8.22,3.13)	0.337
V_15_	0	0	−0.89(−2.90,1.12)	0.343
Heart				
D_mean_	3.59 ± 1.58	3.61 ± 1.51	−0.03(− 0.19, 0.14)	0.718
D_max_	38.39 ± 8.06	38.15 ± 8.01	0.24(−0.34, 0.83)	0.371
V_5_	19.72 ± 13.36	21.67 ± 10.71	−1.93(−7.21,3.33)	0.426
V_25_	0.44(0.04,2.39)	0.50(0.04,2.32)	0.02(−0.09,0.13)	0.663
LAD				
D_mean_	13.35 ± 7.63	13.66 ± 7.29	−0.32(−0.71, 0.07)	0.099
D_max_	25.64 ± 11.03	25.76 ± 10.42	−0.12(−1.17, 0.92)	0.795
V_5_	93.65(68.33,100)	93.05(64.42,100)	−2.30(−4.82,0.22)	0.069
V_30_	0(0,10.01)	0(0,10.52)	−0.05(− 0.41,0.32)	0.786
V_40_	0	0	0.763(−0.96,2.49)	0.343
Left Vessile				
D_mean_	4.91 ± 2.16	4.99 ± 2.12	−0.09(− 0.35, 0.18)	0.482
V_5_	33.08 ± 1172	33.82 ± 11.91	−0.75(−2.90,1.40)	0.451
V_23_	0.28(0.09,5.08)	0.35(0.09,3.88)	0.21(−0.16,0.58)	0.229
Right breast				
D_mean_	1.74 ± 1.09	1.70 ± 1.17	0.04(−0.06, 0.14)	0.410
D_2%_	5.21(3.84,10.36)	5.37(3.48,10.12)	−0.28(−1.43,0.87)	0.597
V_5_	3.74(0.52,9.16)	2.98(0.66,6.46)	0.72(−0.37,1.80)	0.17
Spinal cord				
D_max_	0.32 ± 0.06	0.31 ± 0.06	0.01(0,0.02)	0.121
D_2%_	0.29 ± 0.05	0.28 ± 0.05	0.01(0,0.02)	0.037

**Table 4:** Dosimetric parameters of PTV and OARs for 10 cases of right breast cancer after breast-conserving surgery

Parameter	Plan+	Plan-	$\overline{D}$(95%CI)	*p*
PGTV				
Coverage Index	97.78 ± 1.03	96.28 ± 1.44	1.50(0.59,2.41)	0.004
D_mean_	47.04 ± 0.25	46.98 ± 0.25	0.05(− 0.-3,0.13)	0.178
HI	1.07 ± 0.01	1.07 ± 0.01	−0.001(− 0.0008,-0.01)	0.025
CI	0.65 ± 0.09	0.65 ± 0.10	0(−0.20,0.15)	0.740
PTV				
Coverage Index	95.74 ± 1.96	95.29 ± 0.95	0.46(−0.68,1.59)	0.391
D_mean_	42.71 ± 0.66	42.79 ± 0.57	−0.09(− 0.23,0.05)	0.187
HI	1.16 ± 0.03	1.17 ± 0.03	−0.01(− 0.03,0)	0.093
CI	0.80 ± 0.06	0.82 ± 0.06	−0.02(− 0.03,0)	0.046
Skin				
D_mean_	32.10 ± 1.72	28.85 ± 2.26	3.25(2.65, 3.86)	0.000
V_5_	95.80 ± 3.82	93.86 ± 4.73	1.94(0.78,3.10)	0.004
V_10_	91.56 ± 5.22	88.68 ± 5.80	2.88(1.71, 4.05)	0.000
V_20_	84.09 ± 6.66	79.23 ± 7.64	4.86(2.82, 6.89)	0.000
V_30_	73.03 ± 7.03	61.12 ± 8.05	11.91(10.09, 13.73)	0.000
V_40_	20.50 ± 7.73	9.99 ± 5.38	10.51(6.87,14.14)	0.000
Right Lung				
D_mean_	7.97 ± 1.51	7.80 ± 1.45	−0.03(−0.20, 0.14)	0.718
V_5_	37.34 ± 5.29	37.16 ± 4.45	0.18(−0.99, 1.35)	0.740
V_10_	24.51 ± 4.53	24.46 ± 4.29	0.05(−0.73, 0.84)	0.881
V_20_	14.80 ± 3.97	15.20 ± 3.86	−0.40(− 0.81, 0.01)	0.056
V_30_	8.73 ± 3.65	8.82 ± 3.51	−0.08(− 0.46, 0.29)	0.629
Left Lung				
D_mean_	0.41 ± 0.19	0.39 ± 0.18	0.02(−0.01 0.05)	0.130
V_5_	0	0	0.17(−0.11,0.45)	0.195
V_15_	0	0	0	
Heart				
D_mean_	1.16 ± 0.62	1.15 ± 0.63	0.01(−0.05, 0.06)	0.858
D_max_	7.79 ± 2.94	7.71 ± 2.83	0.09(−0.28, 0.45)	0.610
V_5_	0.23(0.01,2.05)	0.14(0.01,1.46)	0(−0.52,0.52)	0.988
V_15_	0	0	0	
Liver				
D_mean_	4.45 ± 2.84	4.36 ± 2.89	0.09(−0.11, 0.29)	0.349
V_5_	26.74 ± 18.92	27.34 ± 19.43	−0.59(−2.40,1.23)	0.487
V_13_	11.21 ± 10.36	11.20 ± 10.73	0.01(−0.84,0.85)	0.989
Left breast				
D_mean_	1.08 ± 0.77	1.02 ± 0.70	0.06(0, 0.12)	0.050
D_2%_	4.48(2.83,8.85)	3.21(2.70,8.52)	0.74(0.05,1.42)	0.038
V_5_	1.08(0.01,6.86)	0.72(0,5.74)	0.20(−0.10,0.49)	0.167
Spinal cord				
D_max_	0.43 ± 0.11	0.41 ± 0.12	0.02(0,0.04)	0.086
D_2%_	0.39 ± 0.11	0.37 ± 0.10	0.02(0.01,0.04)	0.014

### Dose difference distribution map (plan+ − Plan-)

**Fig. 3:**
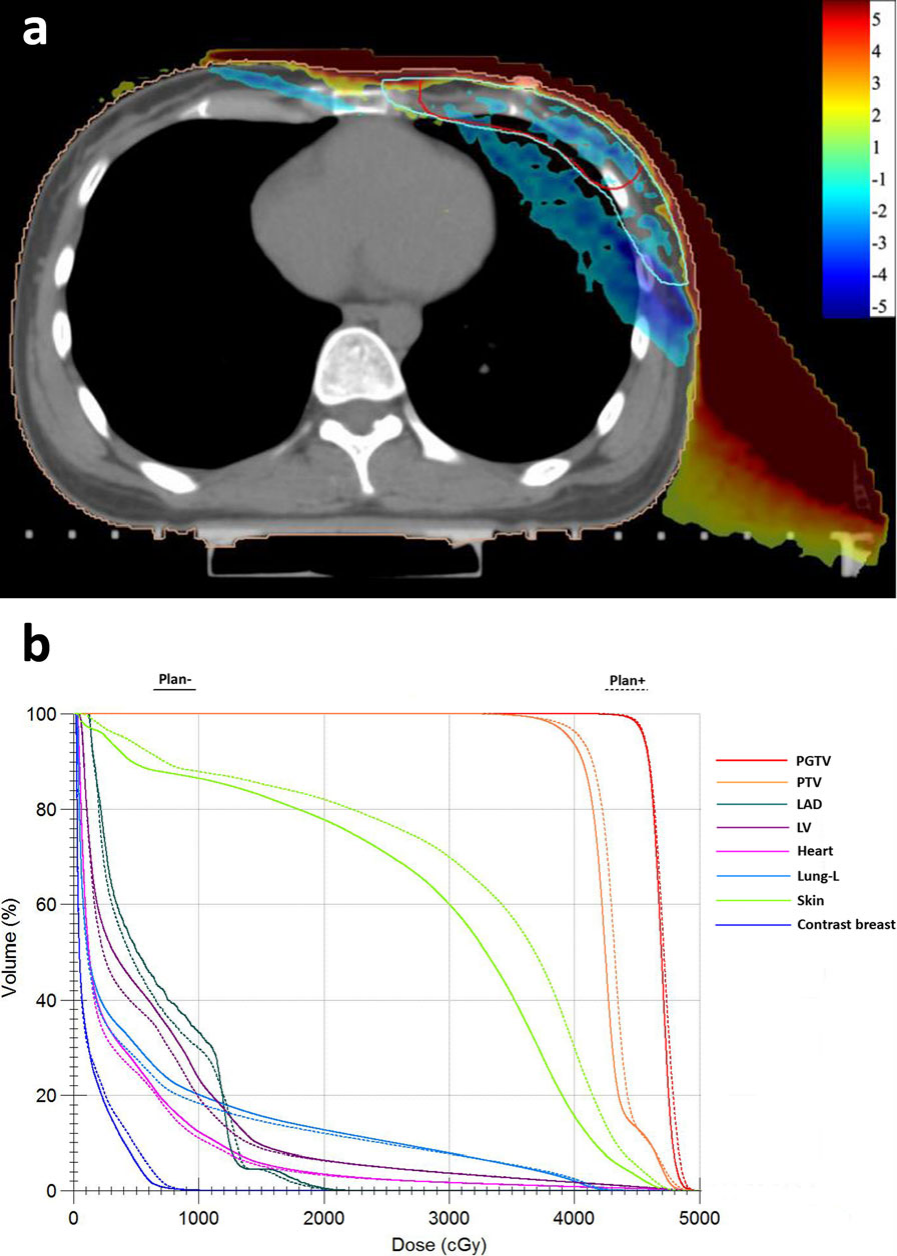
**a** Dose difference distributions of the cross-sectional plane for a typical patient with left-side breast cancer after BCS. The dose difference was calculated by subtracting Plan- (calculated without immobilization devices) from Plan+ (calculated with the whole immobilization devices included in the external body structure). **b** DVH results of Plan- and Plan+ for a typical patient with left-side breast cancer after BCS. The solid and dotted lines represent the results of Plan- and Plan+, respectively

The dose difference distributions were calculated as Plan+ subtracted from Plan-. As shown in Fig. 3a, the blue to red gradient represented different absolute dose values ranging from − 5 to 5 Gy. The build-up effect and radiation scattering caused by the immobilization devices dramatically altered the dose distributions. The skin dose was observably increased in the irradiated region when the immobilization devices were included in the calculations. In other words, the skin dose was underestimated by approximately 6 Gy if the immobilization devices were not included in the external contour. The doses in other regions including Lung-L and PTV were also decreased, a finding similar to the DVH and data comparison results, as show in Fig.3b.

## Discussion

Patient immobilization devices have become an important tool to guarantee the accurate delivery of highly conformal dose distributions [3]. As the materials used in immobilization devices are not completely X-ray transmissible and can cause attenuation of the delivered dose, the dosimetric effects of immobilization devices should be included in dose calculations [17, 24]. Beam attenuation from the couch, additional inserts, and immobilization devices can cause a misrepresentation of the actual dose delivered to the PTV, with a deviation of more than the recommended 3–5% accuracy range reported by Olch [3]. Chen reported the attenuation of head and neck immobilization devices, which reduced the dose coverage rate from 1.51 to 9.92% and the average dose from 0.93 to 1.92% of planning target volumes in nasopharyngeal carcinoma [24]. Olson assessed the dose variance from immobilization devices in volumetric-modulated arc therapy (VMAT) head and neck treatment planning and found that the plan calculated without immobilization devices was problematic, showing compromised V_95_, D_100_, and PTV coverage [17]. However, in our study, we observed no clinically important effect of supine breast immobilization devices on the dosimetric parameters of PTV and PGTV, with a deviation of less than 3%. The potential reason for this difference may lie in the fact that not all radiation beams passed through the couch in our study. Puysseleyr et al. measured the dosimetric impact of a prone breast immobilization device and found that beam attenuation accounted for 7.6% (6 MV X-ray) of beams passing through the couch top-base plate combination and almost 5% for beams traversing the couch-top [7]. Thus, a beam attenuation of less than 3% occurred when the beam passed through the base plate only, similar to our findings.

In addition, the bolus effect cannot be avoided. Beams, especially posterior oblique orientations beams, passing through the immobilization devices involved in treatment can result in increased unexpected skin doses to ultimately affect the dosimetric effects [1, 8, 24, 25]. Kelly et al. utilized radiochromic film and MOSFET detectors to quantify the effect of an immobilization cast on skin dose in breast radiotherapy, and observed an increase in skin dose up to 45.7 and 62.30%, which is similar to our results of patients with breast-conserving surgery ($\overline{D}$_% = 51.86 and 51.63% for left and right BC)_ [1]. Lee and colleagues measured the inguinal region skin dose using thermoluminescent dosimeters (TLDs) in prostate cancer patients. They found that the TLD-measured dose was two-fold higher than the calculated dose that did not contour the vacuum cushion and couch and was similar to the calculated dose with both devices contoured [8]. Chen et al. also reported that, due to the bolus effect of head and neck immobilization devices, the dorsal neck skin dose was significantly increased by approximately 8 Gy (53%) in multi-field IMRT for nasopharyngeal cancer [24]. Similarly, Ali et al. find that thermoplastic mask used to patients for head and neck, pelvis and thoracic treatment can significantly increase skin dose by up to a factor of 4 more than that without the mask using 6 MV beams [25]. Radiation-induced skin toxicity (RIST) is a predominant adverse effect and deserves consideration as severe skin toxicity can lead to treatment cession and cosmetic changes in BC patients. In the present study, we also observed a bolus effect of immobilization devices, as the skin mean dose and volume receiving 5–40 Gy were significantly increased in Plan+. This effect was more obvious in patients after BCS, mainly because the breast was spatially closer to the immobilization device compared to the chest. Moreover, the V_30_ appeared to be the most sensitive parameter except for in patients with right BC receiving radical mastectomy. Pastore et al. reported that breast skin receiving doses ≥30 Gy was the most predictive parameter of acute RIST [26], while Tsair-Fwu showed that skin receiving a dose > 35 Gy (V_35_) was the most significant dosimetric predictor associated with radiation dermatitis grade 2+ toxicity. The higher V_30_ in our study may translate into increased RIST. There is currently no standard of practice to include immobilization devices within body contours; the results of this study showed that the actual skin dose was underestimated when treatment beams passed through the couch top and immobilization devices, which, in turn, induced more and severer dermatitis. Our results indicate that immobilization devices should be included in dose calculations in BC treatment planning and that the skin of the breast region should be delineated as an OAR and that a dose-volume constraint for skin should be defined whenever possible.

Despite the positive results of this study, it has some limitations. A larger patient population, as well as different TPS or calculation algorithms, dosimetry techniques, and dose measurements are required in future studies.

## Conclusions

This study calculated and evaluated the dosimetric effects on the skin of supine immobilization devices for BC in IMRT plans. The data showed a significantly increased skin dose, especially in patients with BCS, with both the V_30_ and V_40_ of the skin increasing sharply by more than 10%. These findings should remind radiation practitioners to pay attention to the skin dose caused by the immobilization devices and to seek solutions to ameliorate these negative effects. It is possible to include the immobilization devices within the external body contour and to account for the skin dose increment in the TPS calculation in BC treatment planning.

## Data Availability

The datasets used and/or analysed during the current study are available from the corresponding author on reasonable request.

## Abbreviations

BC: breast cancer
BCS: breast-conservation surgery
CI: conformity index
CSTRO: China Society for Radiation Oncology
CT: computed tomography
CTV: clinical target volume
Dmean: mean dose
DMLC: Dynamic Multi-Leaf Collimator
DVH: dose-volume histogram
HI: homogeneity index
ICRU: International Commission on Radiation Units and Measurements Reports
IMRT: intensity-modulated radiation therapy
LAD: left anterior descending artery
LV: left ventricle
MC: Monte Carlo
MUs: monitor units
OAR: organ at risk
PTV: planning target volume
RIST: radiation-induced skin toxicity
ROI: region of interest
TLD: thermoluminescent dosimeter
TPS: treatment planning system
VMAT: volumetric-modulated arc therapy
Vx: volume of skin receiving equal to or greater than X Gy
